# Treatment-Resistant Blunted HPA Activity, but Reversible Cardiovascular Stress Reactivity in Young Women With Eating Disorders

**DOI:** 10.3389/fpsyt.2020.00726

**Published:** 2020-07-22

**Authors:** Serkan Het, Silja Vocks, Jutta M. Wolf, Stephan Herpertz, Oliver T. Wolf

**Affiliations:** ^1^ Department of Cognitive Psychology, Faculty of Psychology, Ruhr University Bochum, Bochum, Germany; ^2^ Department of Clinical Psychology and Psychotherapy, Osnabrück University, Osnabrück, Germany; ^3^ Department of Psychology, Brandeis University, Waltham, MA, United States; ^4^ Department of Psychosomatic Medicine and Psychotherapy, LWL-University Clinic, Ruhr University Bochum, Bochum, Germany

**Keywords:** eating disorder, anorexia nervosa, bulimia nervosa, cortisol, hypothalamus–pituitary–adrenal (HPA) axis, stress, Trier Social Stress Test (TSST), alpha amylase

## Abstract

Previous research has provided evidence for a reduced neuroendocrine stress response in women with eating disorders (EDs). In the present study female in-patients with Anorexia and Bulimia nervosa were compared to female healthy controls (HC) before and after completing an in-patient treatment program. Salivary cortisol, alpha-amylase (sAA), heart rate response (HR), high-frequency heart rate variability (HF-HRV) and negative affective state were measured before, during and after exposure to the Trier Social Stress Test (TSST) at pre- and post-treatment. Patients with EDs (*n* = 13) showed significantly less ED symptoms at post-treatment. Compared to HC (*n* = 22), patients displayed a blunted cortisol stress response combined with overall attenuated sAA levels at pre-treatment. At post-treatment, the blunted cortisol stress response was still observable, while the differences in sAA responses disappeared. HR was attenuated at pre-treatment in patients, also indicated by a stronger HF-HRV throughout the TSST. These cardiovascular differences disappeared at post-treatment. Patients reported in general (pre- and post-treatment) more negative affect compared to HC. This study provides further evidences of a hypo-reactive hypothalamus–pituitary–adrenal axis (HPA) in patients with EDs which persists even after symptom recovery while initial low cardiovascular stress reactivity apparently can be restored by psychotherapy. Given the small sample size the findings have to be considered preliminary.

## Introduction

Psychosocial stress is a potential risk factor for mental disorders (1–4). This includes the development and the maintenance of eating disorders (EDs), such that patients with EDs often experience negative life events or chronic stress before disease onset (5–11). Although not specific to patients with EDs, previous research suggests altered stress responsivity as a risk factor or disorder sequel (12–14), with ED patients relatively consistently showing sympathetic nervous system (SNS) and hypothalamus–pituitary–adrenal (HPA) axis activity alterations (15).

More specifically, patients with Anorexia nervosa (AN) and Bulimia nervosa (BN) both show a blunted SNS activity compared to healthy controls (HC) under resting [(16), e.g. (17–19)] and acute stress conditions (17, 20–23). Recently, these SNS findings were confirmed by a meta-analysis (24).

In addition, patients with EDs also show dysregulations of the HPA axis, indicated by higher cortisol in urine, serum or saliva in patients with EDs (21, 23, 25–29). Regarding HPA axis reactivity, we previously reported that patients with EDs show a blunted cortisol response to acute psychosocial stress (19).

Only a few studies exist that investigated whether those neuroendocrine deviations normalize after successful treatment. One study compared heart rate and affective responses to a speech tasks in formerly anorectic, adolescent patients (30). The girls reported higher levels of negative affect, but did not show a blunted heart rate response during the stressor, suggesting a restored SNS response after recovery. Another study, investigated the cortisol awakening response (CAR) in underweight and weight-restored women with AN (31). The underweight patients showed an exaggerated CAR, whereas weight-restored patients had a CAR similar to HC, suggesting that weight gain may help to normalize HPA axis activity in patients with AN.

Together, these findings indicate that low cardiovascular activity and exaggerated CAR can be restored by effective treatment, while others (e.g. negative affect) seem to persist even after weight recovery/treatment. We did not find any studies in patients with AN or BN on the HPA axis reactivity after successful treatment. The aim of the present study was to characterize differences in stress responses between ED patients and HC over time. In-patients with AN or BN were investigated with regard to psychological and physiological responses to a standardized laboratory stressor [Trier Social Stress Test: TSST; (32)], before and after a long-term in-patient treatment program. In line with a transdiagnostic perspective (33, 34), which conceptualizes AN and BN as states on a continuum of psychopathology, patients with AN and BN were investigated as one group [cf. (19, 35, 36)].

Based on previous observations (19, 36) we predicted that patients with EDs will show blunted cortisol stress responses at pre-treatment compared to HCs. As described before (19) we suppose a long-term exhaustion of the HPA axis in patients with ED that makes individuals vulnerable for this and other psychological disorders. Therefore we expected that the HPA axis stress reactivity dysfunction persists in patients with ED even after treatment and leads to a blunted cortisol stress responses in patients compared to HC at post-treatment. Additionally, the patients were expected to improve in SNS responses comparable to HCs at post-treatment as already shown earlier [e.g. (30)]. Current studies showed that the HPA does not habituate, if a stress protocol is repeated weeks later or the stress inducing tasks are slightly altered (37–39). However, these studies used special forms of the TSST, protocols similar to the TSST and a time interval of 24 hours or 10 weeks between the first and the second TSST session. Referring to these studies we predicted similar cortisol and SNS stress responses from pre- to post-treatment for the HC. Lastly, since strong responses to negative social feedback appear to persist beyond recovery in with EDs (30, 40, 41), we predicted a stronger negative affect response to the TSST in ED patients compared to HCs both before and after treatment.

## Methods

### Participants

For the current report, only patients with AN or BN who completed an in-patient treatment and provided complete pre-treatment and post-treatment data were included. Further, as HPA axis dysfunctions are well-described in post-traumatic-stress disorder, borderline personality disorder, and schizophrenia (42, 43), patients with EDs fulfilling the criteria of at least one of those diagnoses were excluded. Participants with previous TSST exposure and participants who did not refrain from physical exercise or eating one hour before testing were excluded as well. In contrast, smoking (44) and use of oral contraceptives or estrogene–progesterone combination medication to prevent low bone density (45, 46) were permitted. All patients were recruited from the Christoph–Dornier Clinic for Psychotherapy in Münster (Germany) and the Department of Psychosomatic Medicine and Psychotherapy, LWL-University Clinic in Bochum (Germany). Both clinics are specialized in the treatment of EDs. Weight restoration, regaining of normal eating habits, and reduction of bulimic symptoms like vomiting, intensive exercises, or abuse of laxative medication were achieved by an in-patient treatment program according to the national guidelines for the diagnosis and treatment of EDs (47). This treatment program contains elements of cognitive behavioral therapy (CBT) and psychodynamic therapy. It aims to restore healthy eating and reaching a healthy body weight by structuring and accompanying the patients during breakfast, lunch and dinner. A personalized treatment plan was created for each patient based on the processes that appear to be maintaining the eating problem. Information about nutrition, cognitive restructuring, mood regulation, psychodynamic conflicts and relationship problems, social skills, body image concern, self-esteem, and relapse prevention were covered.

Participants of the HC group were physically and mentally healthy, medication-free (except of oral contraceptives) female students with a BMI within the normal range (18.5–26 kg/m^2^). They were recruited at the Universities of Bochum and Münster. All participants provided written informed consent. The study protocol was approved by the institutional review board of the Faculty of Psychology, Ruhr University Bochum.

A total of 28 patients (range of age: 15–46) and 26 healthy controls (range of age: 18–37) were recruited (19). For the current study, complete data are available for 13 patients (range of age: 18–29) and 22 healthy controls (range of age: 18–46). In more detail, at pre-treatment, *n* = 18 in-patients with AN and *n* = 10 in-patients with BN were recruited who fulfilled the diagnostic criteria of AN or BN according to the 4th edition of the Diagnostic and Statistical Manual of Mental Disorders (DSM-IV-TR, 2000). Ten AN patients and five BN patients terminated the treatment program or their participation in the study before the second assessment and thus had to be excluded from analyses, resulting in a final patient sample of *n* = 13 (AN: *n* = 8, BN: *n* = 5). ED patients were diagnosed on average shortly before entering treatment. Of the initial 26 healthy female control participants, *n* = 3 dropped out of the study and *n* = 1 participant had to be excluded due to weight loss resulting in a BMI <18.5 kg/m^2^.

### Trier Social Stress Test (TSST)

The TSST was performed before and after the intervention as described by Kirschbaum et al. (32). It has been shown to be effective in eliciting HPA axis and SNS responses and negative affect (48–50). In short, participants were asked to convince a panel of judges that they were the perfect candidate for a job opening in their ‘dream occupation’. After a five minute preparation period, participants were asked to talk for a duration of 5 min exclusively about job-relevant personality traits while refraining from reciting application material information. If the participant finished her speech in less than 5 min, pre-formulated questions were asked. During the subsequent 5 min, the participants were asked to count backwards in steps of 17 from 2,043 as fast and as accurately as possible. Whenever the participant made a mistake, she had to start over at 2,043. Both members of the committee were dressed in white lab coats and acted in a neutral manner. All participants had to attend to the TSST before and after treatment. To reduce habituation, the post-treatment TSST was conducted by different panel members and a different subtraction task was used (counting backwards in steps of 27 from 3,074). No other changes were made to the TSST protocol.

### Saliva Sampling and Biochemical Analyses

Saliva samples were obtained using Salivette sampling device (Sarstedt, Nümbrecht, Germany) to assess free salivary cortisol and salivary alpha-amylase (sAA) as HPA axis and SNS markers, respectively (51). Samples were collected one minute before (−1) and one (+1), ten (+10) and twenty-five (+25) minutes after each TSST. Cortisol concentrations were measured using a commercially available immunoassay with chemiluminescence-detection (IBL-Hamburg, Germany). Salivary alpha-amylase activity was measured using a quantitative enzyme kinetic method, as described earlier (51). Inter- and intra-assay coefficients of variation were below 10% for both assays.

### Autonomic Assessment

HR and HRV were assessed as indicators of autonomic changes using the Polar watch system (RS800CX, Polar, Finland) for heart-beat monitoring. This system has been shown to have high reliability and validity (52, 53). Spectral analysis of HRV was performed with the Polar Pro Trainer 5 Professional Training Software, based on inter-beat intervals (R-R intervals). The software extracts HRV in various frequency bands and expresses it as ms^2^. Frequency domain variables were derived from HR measurements during a time span of 5 min before the TSST (baseline) and during the first 5 min of the TSST (54). HRV was assessed for both TSSTs, pre- and post-treatment. We concentrated only on high-frequency HRV (HF-HRV, 0.15–0.4 Hz) as it is thought to reflect cardiac vagal function by representing the respiratory sinus arrhythmia, thus indicating parasympathetic activity.

### Assessment of Affect

Participants filled out the *Positive and Negative Affect Schedule* [PANAS; (55, 56)] at arrival at the laboratory, shortly before and immediately after cessation of the TSSTs and 10 and 25 min after the TSSTs. The PANAS is a reliable and valid measure for current affective state (57). It consists of 10 items for positive (e.g., interested, enthusiastic) and negative affect (e.g., upset, ashamed) which are rated on a five-point scale (1 = “very slightly or not at all”, 5 = “extremely”). Average scores for positive and negative affect were calculated.

### Procedure

All participants underwent a diagnostic examination using the German versions of the *Structured Clinical Interview for DSM-IV, Axis 1* [SCID-I; (58)], the *Beck-Depression-Inventory* [BDI; (59)], the *Symptom-Checklist-90-Revised* [SCL90-R; (60)] and the *Eating Disorder Examination-Questionnaire* [EDE-Q; (61)]. The SCID was administered by trained clinical psychologists. All patients were in-patients and were offered study participation the day of or the day after admission. The patient data were collected after admission to the clinic and before the start of treatment (pre-treatment), and between the end of treatment and before discharge from the clinic (post-treatment). Assessment times for HC were synchronized. The second data collection took place on average 8.47 (SEM: ± 1.8) weeks (range: 2–58 weeks) after the first assessment.

Each assessment involved two days of participation, for a total of four study days. On the first pre-treatment study day, participants provided informed consent as well as information pertaining to their menstrual cycling phase and responses to questionnaires. On the second day, participants were exposed to the TSST in the hospital. The TSST was administered in the afternoon between 2pm and 5pm to minimize between-participant variation in pre-TSST baseline cortisol levels (62). After arrival at the lab, participants were seated in a quiet room. The Polar watch was fitted and started to record HR and HRV continuously until the end of the test day. Additionally, participants answered the PANAS for the first time. Subsequently, a first saliva sample was obtained and the PANAS was answered for the second time. Next, the participants were exposed to the TSST and immediately afterwards, a post-TSST saliva sample (+1 min) and PANAS self-report were collected. Two more (+10 min, +25 min) saliva samples and PANAS self-reports were obtained subsequently. This procedure was repeated at post-treatment, except for omission of informed consent. A debriefing of the TSST was conducted at the end of post-treatment assessment only.

### Statistical Analyses

Demographic and descriptive variables were investigated for group differences and changes over time by Pearson’s Chi-square test, Student’s *t*-test for independent samples and paired-samples *t*-test, respectively. All data were tested for normal distribution with Kolmogorov–Smirnov test (K–S test). In case of a significant K–S test, data were log-transformed and the subsequent statistical analyses were performed with the transformed data. Area under the curve [AUC, (63)] was calculated with respect to increase (AUCi) for the neuroendocrine variables (cortisol and sAA). Cortisol responders to the TSST were defined as showing a 1.5 nmol cortisol increase between baseline and 10-minute post-stress value as recommended (64). Analyses of variance (ANOVA) for repeated measures were performed on cortisol and sAA levels, on PANAS scores, HR, and HRV to reveal possible effects of time (e.g., four cortisol time points), group (ED, HC), treatment (pre-treatment, post-treatment) and the respective 2-way interaction and 3-way interaction. Greenhouse–Geisser adjusted *p*-values are reported in case of violation of the sphericity assumption. Due to potential effects on HPA axis reactivity, analyses pertaining to cortisol indices were repeated controlling for smoking, use of oral contraceptives and disease duration (62), i.e., univariate and repeated-measures ANCOVAs were computed. Statistical analyses were performed using IBM SPSS 25 (Chicago, IL) for Mac OS X. Level of significance was defined as *p* <.05. Effects with *p* <.10 were interpreted as trends.

## Results

### Sample Characteristics

Sample characteristics are summarized in **Table 1**. Patients with EDs and HC did not differ in age and body height, while BMI was significantly lower in patients both at pre-treatment (*t_33_* = 4.9, *p* < .001) and post-treatment (*t_33_* = 2.8, *p* = .008). Comparing pre- and post-treatment BMI and EDE-Q scores in patients only, however, revealed that ED patients showed a significant increase in weight (*t*_12_ = 4.8, *p* < .001) and a significant reduction in ED symptoms (*t*_9_ = 4.3, *p* = .002), reflecting treatment success. The two groups did not differ in smoking behavior, average cigarettes per day, average days between pre- and post-treatment testing, menstrual cycle phase, or intake of oral contraceptives (all *p*s >.05).

**Table 1:** Means and standard error of means for different descriptive variables for each group.

Variable	ED Patients(n = 13)	HC(n = 22)	*t*-test
Age (years)	21 ( ± 1.3)	23.1 ( ± 1.1)	*t_33_* = 1.2, *p* = .22
Height (cm)	165 ( ± .01)	169 ( ± .01)	*t_33_* = 1.7, *p* = .10
Weight (kg):			
pre-treatment*	47.1 ( ± 2.5)	62.1 ( ± 1.9)	*t_33_* = 4.8, *p* <.001
Patients with AN	42.5 ( ± 2.8)		
Patients with BN	54.5 ( ± 2.4)		
post-treatment*	53.5 ( ± 2.0)	62.3 ( ± 1.9)	*t_33_* = 3.0, *p = .*005
Patients with AN	51.4 ( ± 2.7)		
Patients with BN	56.9 ( ± 2.9)		
BMI (kg/m^2^):			
pre-treatment*	17.2 ( ± .80)	21.9 ( ± .60)	*t_33_* = 4.9, *p* <.001
Patients with AN	15.5 ( ± .75)		
Patients with BN	19.8 ( ± .48)		
post-treatment*	19.5 ( ± .52)	21.9 ( ± .59)	*t_33_* = 2.8, *p* = .008
Patients with AN	18.7 ( ± .61)		
Patients with BN	20.8 ( ± 64)		
EDE-Q:			
pre-treatment*	4.3 ( ± .29)	.61 ( ± .11)	*t_26_* = −12.5, *p* <.001
post-treatment*	2.9 ( ± .36)	.49 ( ± .12)	*t_27_* = −8.0 *p* <.001
Smoking (n):	4	4	$\chi_{1}^{2}$ = .73, *p* = .40
cigarettes per day	10.5 ( ± 2.1)	7.6 ( ± 6.0)	*t*_7_ = −.81, *p* = .45
Days between testing	56.4 ( ± 16.8)	64.8 ( ± 18.2)	*t_32_* = .30, *p* = .76
Weeks between testing	7.7 ( ± 2.4)	8.91 ( ± 2.6)	*t_32_* = .30, *p* = .75
Menstrual cycle (n):			
Pre-treatment Follicular Luteal	0 1	2 10	$\chi_{1}^{2}$ = .19, *p* = .66
Post-treatment			
Follicular Luteal	1 3	6 10	$\chi_{1}^{2}$ = .22, *p* = .64
Oral Contraception (n)	7	6	$\chi_{1}^{2}$= .002, *p* = .97

Asterisks indicate a significant group difference.

### Cortisol Stress Response

**Figure 1:**
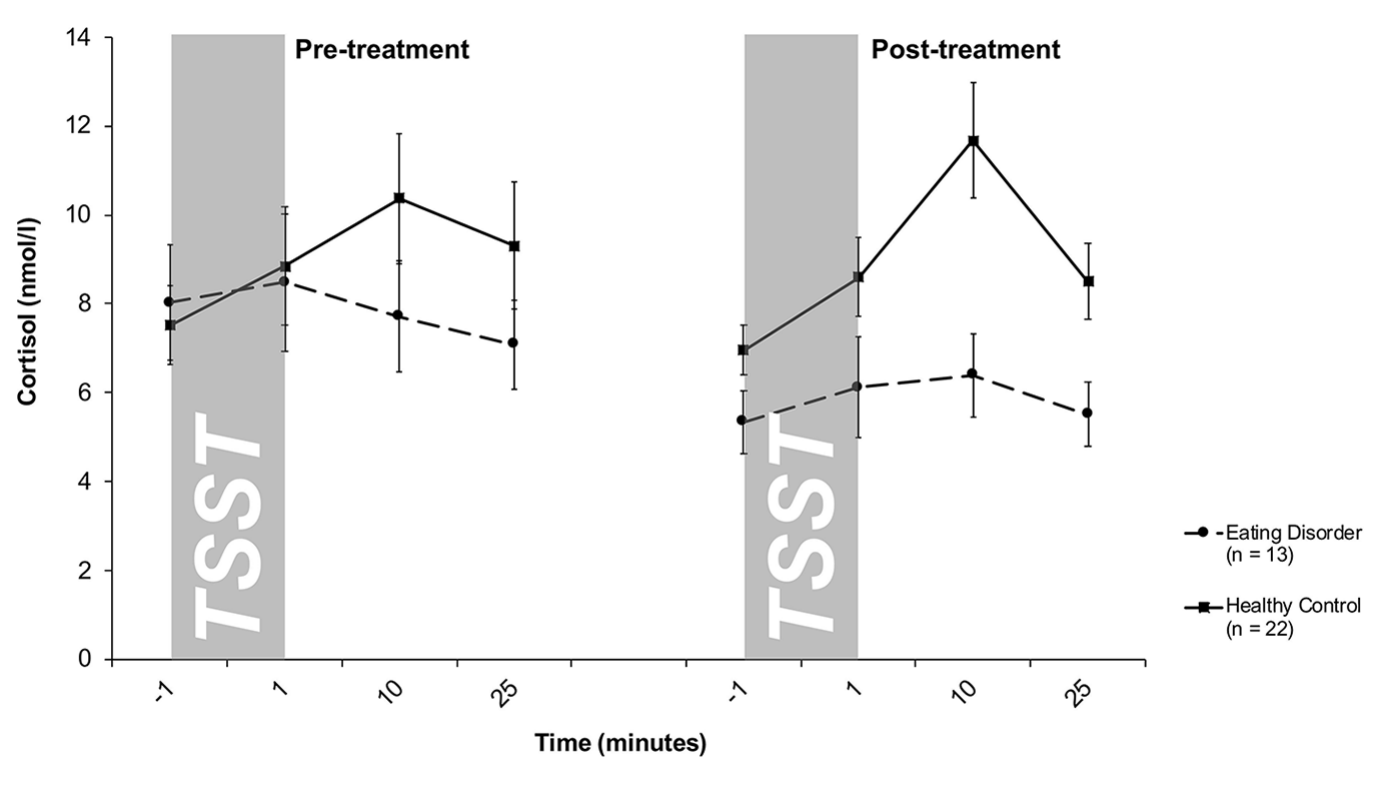
Cortisol stress responses (means and standard errors) pre-treatment (left) and post-treatment (right) in patients with eating disorders and healthy controls (HC). At both pre- and post-treatment, only HC responded to the TSST with increases in salivary cortisol levels.

Salivary cortisol levels are shown in **Figure 1**. A mixed 2 × 4 × 2-factorial ANOVA for repeated measures with *Treatment* (2) and *Time* (4) as within-subjects factor and *Group* (2) as between-subjects factor revealed a significant effect of Time (*F*_3/99_ = 7.1, *p* < .001) and Group (*F*_1/33_ = 2.7; *p* = .10), as well as a significant Time-by-Group interaction (*F*_3/99_ = 5.1; *p* = .004), reflecting an increase in cortisol levels in response to the TSST in the HC group and a blunted response observed in ED patients. This pattern persisted over time, indicated by a lack of Treatment-related effects (Treatment: *F*_1/33_ = 1.5; *p* = .27; Treatment-by-Time: *F*_3/99_ = 1.6; *p* = .19; Treatment-by-Group: *F*_1/33_ = 1.3; *p* = .26; Treatment-by-Time-by-Group: *F*_3/99_ = .51; *p* = .67). This finding was further corroborated by a mixed 2 × 2-factorial ANOVA for repeated measures on AUCi values with *Treatment* (2) as within-subject factor and *Group* (2) as between-subject factor. This analysis revealed a significant main effect of Group (*F*_1/33_ = 5.8, *p* = .02), indicating higher cortisol output above baseline (AUCi) in response to the TSST in HCs compared to ED patients, and again no treatment-related effects (Treatment: *F*_1/33_ = 1.6, *p* = .22; Treatment-by-Group: *F*_1/33_ = .01; *p* = .91). Of note, the significant Time-by-Group interaction on repeatedly-measured salivary cortisol concentrations was even seen when smoking (*F*_3/96_ = 4.2, *p* = .008), menstrual cycling phase (*F*_3/96_ = 4.1, *p* = .008), comorbidity with depression (*F*_3/93_ = 4.2, *p* = .008), duration of illness in months (*F*_3/93_ = 3.8, *p* = .01*)* or symptom severity as measured by the EDE-Q (*F*_3/75_ *=* 3.6, *p = .*02) were considered as covariates or the patients were divided into sub-groups according to their differential diagnosis (*F*_6/96_ = 2.9, *p* = .01). Specifically, neither patients with AN nor patients with BN showed a cortisol response to the TSST at pre- and post-treatment.

### Alpha Amylase Stress Response

**Figure 2:**
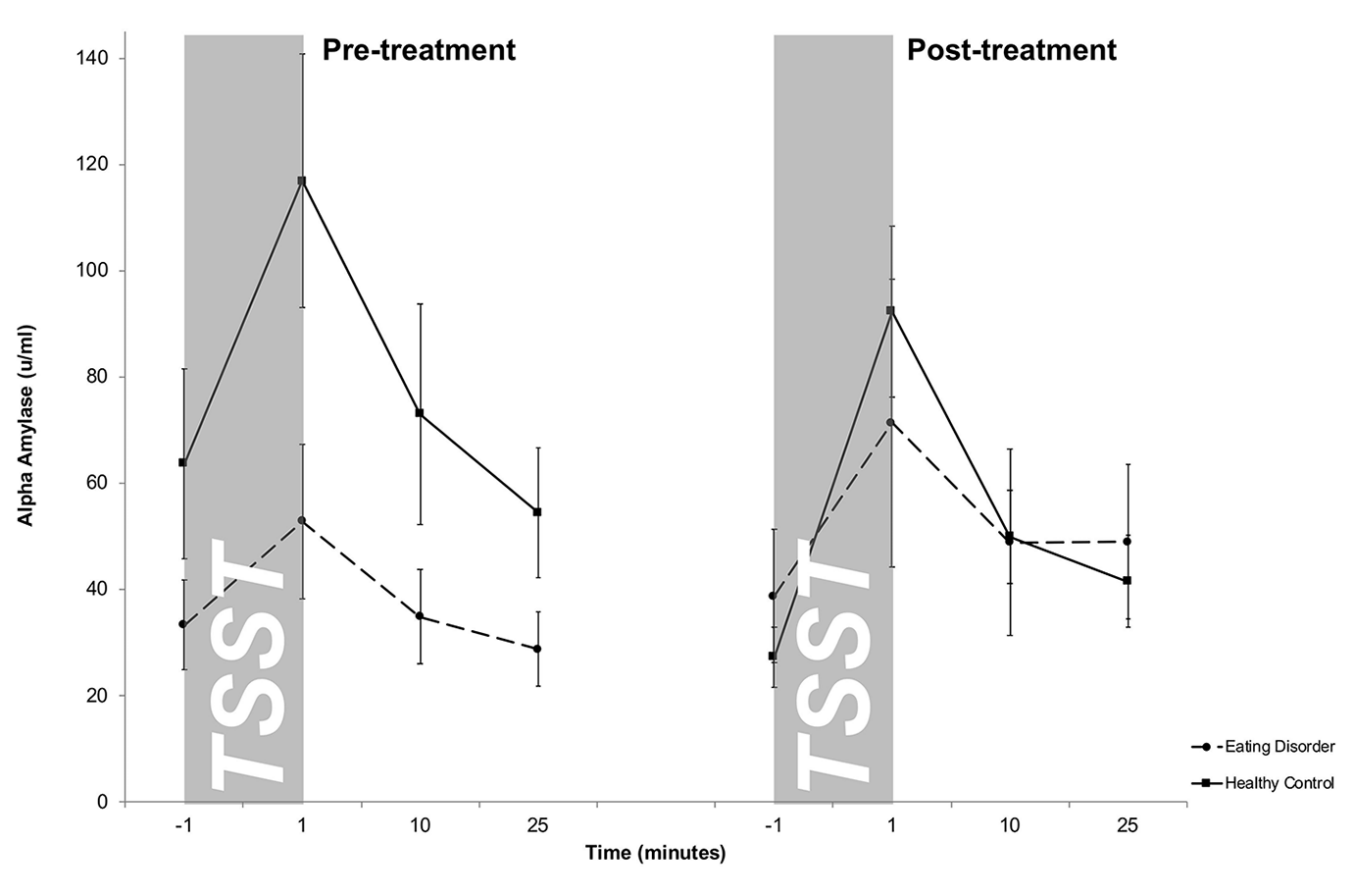
Salivary alpha-amylase (sAA) stress responses (means and standard errors) pre-treatment (left) and post-treatment (right) in patients with eating disorders and healthy controls (HC). While HCs showed an overall stronger salivary alpha amylase output pre-treatment, both groups displayed similar sAA responses post-treatment. For illustrative purposes raw values instead of log-transformed values are shown.

Salivary AA levels are presented in **Figure 2**. The mixed 2 × 4 × 2-factorial ANOVA for repeated measures revealed a significant main effect of Time (*F*_1.4/39.5_ = 18.3, *p* <.001) and a significant Treatment-by-Group interaction effect (*F*_1/27_ = 4.0, *p* = .05), indicating changes in differences between both groups between pre- and post-treatment. Specifically, at pre-treatment, overall higher sAA levels were obtained in HCs compared to ED patients, while both groups showed comparable sAA responses at post-treatment. There were no further interaction effects (Time-by-Group: *F*_1.4/39.5_ = 2.2; *p* = .13; Treatment-by-Time: *F*_3/81_ = 2.0; *p* = .12; Treatment-by-Time-by-Group: *F*_3/81_ = .47; *p* = .70) as well as no additional main effects (Group: *F*_1/27_ = 1.0; *p* = .31; Treatment: *F*_1/27_ = 2.1; *p* = .65). This result was confirmed by a mixed 2 × 2-factorial ANOVA for repeated measures on AUCi values with *Treatment* (2) as within-subject factor and *Group* (2) as between-subject factor revealing a main effect of treatment (*F*_1/26_ = 4.3; *p* = .04), indicating higher AUC_i_ in HCs in contrast to patients at pre-treatment and a missing group difference at post-treatment. The slight decrease in peak sAA levels from pre- to post treatment within HCs turned out to be not significant (*t_18_* = −1.4, *p* = .17) on AUCi.

### Heart Rate Response and Variability

The descriptive results of HR and HRV are presented in **Table 2**. Using a mixed 2 × 2 × 2 ANOVA for repeated measures with *Treatment*, *Time* and *Group* as factors, we found for HR a significant main effect of Treatment (*F*_1/25_ = 7.4; *p* = .01), Time (*F*_1/25_ = 64.8; *p* < .001), Treatment-by-Group interaction (*F*_1/25_ = 7.5; *p* = .01), Time-by-Group interaction (*F*_1/25_ = 5.4; *p* = .03), and a trend for the Treatment-by-Time-by-Group interaction (*F*_1/25_ = 3.7; *p* = .06). These findings suggest an increase in HR response strength from pre-treatment to post-treatment in patients with EDs, while the HR-responses for the HCs remained stable. Post-hoc analyses confirmed that patients showed lower HR at pre-treatment during baseline (*t*_28_ = 4.7, *p* < .001) and TSST (*t*_28_ = 1.7, *p* = .05) than HC, although the group difference during the TSST failed to reach Bonferroni corrected level of significance (*p* = .0125). During post-treatment, HR results approximated between both groups during baseline (*t*_28_ = 1.5, *p* = .15) as well as during the TSST (*t*_28_ = 2.0, *p* = .06). Additionally, patients with EDs showed overall higher vagal activity as assessed by HF-HRV indicated by a significant Group difference (*F*_1/25_ = 4.2; *p* = .05) and a trend for the Treatment-by-Group interaction effect (*F*_1/25_ = 2.9; *p* = .10). Except of Time (*F*_1/25_ = 8.9; *p* < .01) there was no further effect (all *p*s > .05).

**Table 2:** Means and standard error of means and the results of ANOVA for repeated measures of heart rate response and the two components of heart-rate variability (HRV).

Cardiac Marker	Measurement	Eating Disorder	Healthy Control
**Heart Rate Response**	**Pre-treatment**		
**(beats/min)**	Baseline	63.5 ( ± 3.1)	80.4 ( ± 2.1)
	Stress	90.2 ( ± 6.0)	100.9 ( ± 3.5)
	**Post-treatment**		
	Baseline	75.7 ( ± 3.8)	81.6 ( ± 2.1)
	Stress	117.0 ( ± 10.3)	98.8 ( ± 3.8)
**HF-HRV (ms^2^)**	**Pre-treatment**		
**(vagal activity)**	Baseline	2609.4 ( ± 774.0)	793.7 ( ± 161.6)
	Stress	1475.2 ( ± 608.8)	455.3 ( ± 57.4)
	**Post-treatment**		
	Baseline	1299.9 ( ± 611.1)	821.4 ( ± 196.6)
	Stress	781.9 ( ± 282.9)	602.8 ( ± 282.9)

### Affective Stress Response

**Figure 3:**
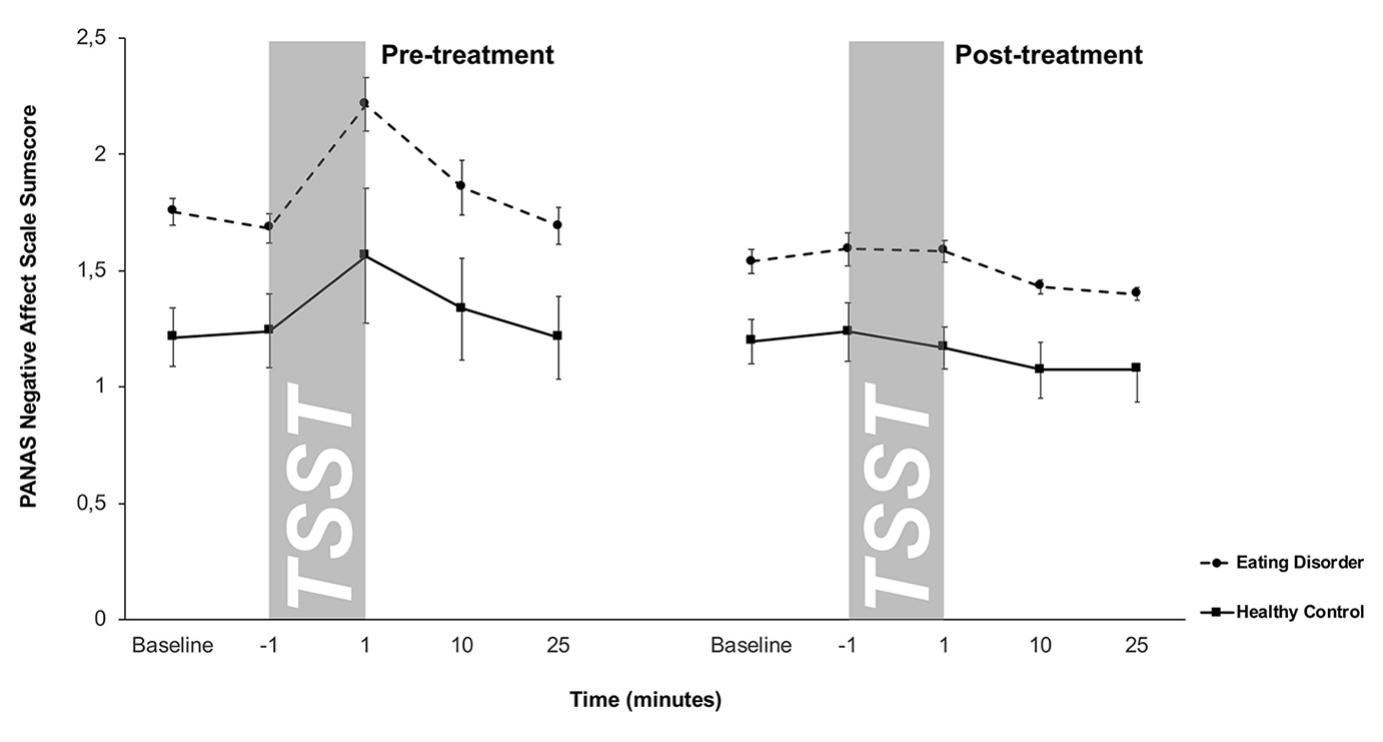
Responses in negative affect to stress (means and standard errors) pre-treatment (left) and post-treatment (right) in patients with eating disorders and healthy controls (HC). Patients with eating disorders showed significantly higher negative affect scores both pre- and post-treatment, while both groups displayed similar blunted negative affect responses at post-treatment.

Negative affect scores of the PANAS are shown in **Figure 3**. A 2 × 5 × 2-factorial ANOVA for repeated measures with *Treatment*, *Time*, and *Group* as factors on the negative affect scale of the PANAS showed a significant main effect of Treatment (*F*_2/32_ = 13.2, *p* = .001), Time (*F*_2.1/68.3_ = 8.8, *p* < .001), Group (*F*_1/32_ = 18.3, *p* < .001) and a significant Treatment-by-Time interaction effect (*F*_1.8/56.04)_ = 6.6, *p* = .004) (all other effects p > .05). Together, this suggested that ED patients showed higher negative affect scores both at pre- and post-treatment compared to HCs. Compared to pre-treatment, post-treatment responses were less dynamic in both groups. HCs showed on a trend level a lower increase in negative affect after TSST at post-treatment compared to pre-treatment (*t*_21_ = 1.6, *p* = .1).

## Discussion

The present study investigated the neuroendocrine, cardiovascular, and emotional stress response to a psychosocial laboratory stressor in patients with EDs before and after an in-patient treatment program. To our knowledge, this is the first study in which pre- and post-treatment effects on stress reactivity were investigated in patients with AN and BN and compared to responses in HCs. We observed the following patterns:

1. In-patients with EDs showed a blunted cortisol stress response to the TSST before as well as after a treatment program compared to HCs. There was no evidence for cortisol stress response habituation in HCs.
2. In contrast to HC, patients showed significantly lower sAA levels, lower HR, and higher HF-HRV before treatment. These differences were diminished at the end of the treatment due to increases in patients’ response strength and lack of habituation in HCs.
3. Patients with EDs reported overall more negative affect compared to HC. This was during pre-treatment, when all participants showed a significant increase in negative affect responses to the TSST, as well as post-treatment, when responses were blunted in both groups.

Although ED patients gained weight and reported reduced core symptoms of AN and BN after treatment, the blunted cortisol response to the TSST in ED patients observed at pre-treatment persisted after treatment. This indicates that HPA axis dysfunctions in these patients may not recover along with disease symptom reduction which is in line with findings in patients with BED as well (65).

This raises the question whether patients with AN and BN may suffer from a long-term exhaustion of the HPA axis due to rigid fasting being a constant metabolic trigger for cortisol release (66, 67). This exhaustion would then become particularly apparent when a patient’s HPA axis is challenged by exposure to a psychosocial stressor. Alternatively, blunted HPA axis reactivity may also precede the development of an ED, i.e., represent a pre-morbid vulnerability factor (16, 17). This would explain why HPA axis reactivity dysfunctions are not affected by treatment. Recently, Monteleone et al. (68) found that childhood trauma-exposed ED patients show significantly reduced stimulated HPA axis activities in contrast to non-trauma-exposed patients, suggesting that early experiences affect the reactivity of the HPA axis in patients with ED. It was also shown in outpatients with AN and BN with an average BMI in the normal range that heightened TSST-induced cortisol secretion is associated with high attachment anxiety or avoidance which might result from childhood trauma (35). Vaz-Leal et al. (36) demonstrated also blunted cortisol stress responses to the TSST in patients with AN and BN (considered as a single group in contrast to HCs) which is in line with our findings. Additionally, they showed that this blunted cortisol response pattern is mainly associated with binge-purging eating behavior as seen typically in BN but sometimes in AN as well. Of note, current studies did not assess long-term effects on HPA axis reactivity. Thus particularly studies in patients for whom treatment success persists over months and years are needed [e.g. (6, 7, 69)]. Such studies would help differentiate between delayed HPA axis recovery and persistence of HPA axis reactivity alterations.

Our results of a blunted cortisol response to the TSST at pre- and post-treatment do not correspond with the results of a recent meta-analysis (24), which showed that patients with EDs and HC do not differ in cortisol stress levels, neither before nor after exposure to an interpersonal laboratory stress task. However, in the meta-analysis, the tests of heterogeneity for cortisol levels before and after a stress task were significant, indicating that the included studies varied markedly in terms of the observed cortisol stress response. These variations might be due to differences between studies with regard of the samples, the methods of cortisol assessment, and in sample sizes. Future studies should use more standardized protocols to measure cortisol responsivity to provide the opportunity for future meta-analyses with more homogeneous studies (50, 70).

Another interesting issue is the response of HC to the second TSST. At post-treatment, the cortisol reactivity of HC was comparable to the results at pre-treatment, i.e. under the conditions of our study there was no habituation to the TSST. This finding is in line with the results of Petrowski et al. (39) who exposed healthy participants to the TSST four times with an interval from 24 h to ten weeks. Although the authors reported decreases in the cortisol responses within 24 h, there were no differences in the HPA response ten weeks later. Other studies using stress protocols similar to the TSST found similar results (37, 38). Usually, it was sufficient if the tasks were slightly altered, e.g. by replacing the committee, changing the topic of the free speech, or the arithmetic task, as we did. These small alterations seem to reduce the predictability of the task and thus, increase the uncontrollability, which, in turn, is a trigger of HPA activity (49). Thus the TSST can be used at different times in the same participants as far as there is a sufficiently long time span between the testing sessions and some alterations in the to be conducted tasks are implemented.

We observed a diminished cardiovascular activity seen at pre-treatment and measured by sAA, HR and HF-HRV in patients with EDs compared to HC. This finding is in line with previous reports of blunted cardiovascular stress reactivity in ED (24). Our data further support the idea that post-treatment, recovered AN and BN patients show a cardiovascular stress reactivity similar to that of HC. As such, our results expand the finding of Miller et al. (30) to include patients with BN. Taken together, it appears that—in contrast to persistent HPA axis dysregulation—blunted cardiovascular stress reactivity can be restored alongside of ED symptom reduction.

As expected, for all assessed cardiovascular parameters, we found no habituation upon second stress exposure in HC. There are studies that used the TSST or TSST-similar stress protocols that showed no significant signs of habituation in salivary alpha-amylase, HR and HRV, if the same protocol is repeated in intervals of three to ten weeks (37, 38, 71). Our data in HC confirm these findings suggesting that the SNS shows uniform activation patterns in response to repeated exposure to psychosocial stress.

Finally, our data show that the higher negative affective state in patients with EDs compared to HC before treatment remained even after significant weight gain and restoration of eating behavior. The meta-analysis of Monteleone et al. (24) also confirmed that patients with EDs have greater negative affect before and after attending a stress protocol. This effect is discussed in light of high attachment anxiety or avoidance (35). Together these observations indicate that the affective response in patients with EDs remains stress-sensitive and negative even after successful treatment.

Interestingly, comparing pre- and post-treatment negative affect independently of group revealed that participants did not show any negative affective response to the TSST at post-treatment. We interpret this result as a sign of psychological habituation to the TSST. Participants were already familiar with this stress protocol at post-treatment and therefore, the test might have lost its surprising effect of novelty and seem to the participants predictable and therefore controllable (38, 49).

To our knowledge, this is the first study that investigated neuroendocrine, cardiovascular and psychological stress responses in patients with EDs before and after an in-patient treatment program and that used the TSST, as already done in other stress related disorders (72–74). The main limitation of our study is the small sample size, especially within the patients group. This threatens the external validity of our study and general conclusions should be drawn cautiously. Our results should be seen as preliminary results and need to be replicated. Indeed, the meta-analysis of Monteleone et al. (24) revealed that studies investigating patients with EDs are often based on small sample sizes. One reason might be the low prevalence of EDs. In Germany a 12-month prevalence of 1.1% for AN and 0.3% for BN in adult women is given (75). This makes it difficult to recruit patients. Beside of this, patients with EDs show a low self-esteem (5). Researchers need to be patient and cautious to motivate these patients to participate in studies with a social evaluation stress task. In sum, future studies are warranted confirming these findings in a larger sample. Additionally, it would be desirable to proof these results across patient groups when separated by ED diagnosis. Further, longitudinal studies with more frequent assessment time-points (e.g. before, during and after treatment) along with expanded assessments until at least 6 months after completion of treatment would provide further valuable insights. To clarify whether the observed HPA changes are a cause or a consequence of the ED, comparable studies are needed in high risk populations (e.g., young women on a diet), ill patients, patients currently under treatment, freshly recovered patients, and long-term recovered patients. Finally, assessment of eating behavior (e.g., daily caloric intake) and gonadal steroids are recommended for future studies to identify potential moderator and mediator variables.

Taken together the present study provided further evidence of a hypo-reactive HPA axis and enhanced negative affect response in patients with AN and BN. It could be demonstrated that both alterations persist even after successful treatment. In contrast, we show that low cardiovascular stress reactivity can be restored in patients with EDs after treatment, suggesting a differential response dynamic of the SNS versus HPA axis. Additionally, it was shown that a repeated TSST presentation is a promising tool to evaluate and detect alterations in stress responsive systems. If further studies confirm our preliminary results and show also that a hypo-reactive HPA axis and an enhanced negative affect stress response are typical patterns or a pre-morbid vulnerability factors for ED, this could help to understand the complex etiology of EDs. This could pave the way to detect high risk individuals and offer them early treatment programs as prophylaxis. Therapeutic interventions that help to restore a normal HPA axis response pattern in these patients need to be developed.

## Data Availability Statement

The datasets generated for this study are available on request to the corresponding author.

## Ethics Statement

The studies involving human participants were reviewed and approved by Faculty of Psychology—Ethics committee, Ruhr University Bochum. The patients/participants provided their written informed consent to participate in this study.

## Author Contributions

SeH: Design, organization and conduction of the study. The paper was written mainly by SeH. SV: Support and recommendations for the design of the study and the recruitment of patients. Additionally SV also read and corrected the final version of the paper. JW: The neuroendocrine data were analyzed in her laboratory. Additionally, JW also read and corrected the final version of the paper especially with regard to the language StH: Support and recommendations for the design of the study and the recruitment of patients. Additionally StH also read and corrected the final version of the paper OW: Financial Support and recommendations for the design of the study. Additionally OW also read and corrected the final version of the paper.

## Funding

We acknowledge support by the DFG Open Access Publication Funds of the Ruhr-Universität Bochum. This work was also supported in part by a young investigator grants from the rectorate of the Ruhr University Bochum awarded to SeH.

## Conflict of Interest

The authors declare that the research was conducted in the absence of any commercial or financial relationships that could be construed as a potential conflict of interest.
